# Loop-mediated isothermal amplification (LAMP) assays for the species-specific detection of *Eimeria *that infect chickens

**DOI:** 10.1186/1746-6148-7-67

**Published:** 2011-11-03

**Authors:** Christopher P Barkway, Rebecca L Pocock, Vladimir Vrba, Damer P Blake

**Affiliations:** 1 Royal Veterinary College, Department of Pathology and Infectious Diseases, University of London, Hawkshead Lane, North Mymms, AL9 7TA, UK; 2 BIOPHARM, Research Institute of Biopharmacy and Veterinary Drugs, a.s., Pohori-Chotoun, Jilove u Prahy 254 49, Czech Republic

## Abstract

**Background:**

*Eimeria *parasites can cause the disease coccidiosis in poultry and even subclinical infection can incur economic loss. Diagnosis of infection predominantly relies on traditional techniques including lesion scoring and faecal microscopy despite the availability of sensitive molecular assays, largely due to cost and the requirement for specialist equipment. Despite longstanding proven efficacy these traditional techniques demand time and expertise, can be highly subjective and may under-diagnose subclinical disease. Recognition of the tight economic margins prevailing in modern poultry production and the impact of avian coccidiosis on poverty in many parts of the world has highlighted a requirement for a panel of straightforward and sensitive, but cost-effective, *Eimeria *species-specific diagnostic assays.

**Results:**

Loop-mediated isothermal amplification (LAMP) is an uncomplicated, quick and relatively inexpensive diagnostic tool. In this study we have developed a panel of species-specific LAMP assays targeting the seven *Eimeria *species that infect the chicken. Each assay has been shown to be genuinely species-specific with the capacity to detect between one and ten eimerian genomes, equivalent to less than a single mature schizont. Development of a simple protocol for template DNA preparation from tissue collected post mortem with no requirement for specialist laboratory equipment supports the use of these assays in routine diagnosis of eimerian infection. Preliminary field testing supports this hypothesis.

**Conclusions:**

Development of a panel of sensitive species-specific LAMP assays introduces a valuable new cost-effective tool for use in poultry husbandry.

## Background

The *Eimeria *species are obligate intracellular protozoan parasites which cause the enteric disease coccidiosis in all livestock species, most notably poultry [1]. The cost of eimerian infection is difficult to quantify, but has been predicted to exceed £1,500 million per annum worldwide [1]. *Eimeria *have an enzootic distribution and both clinical and subclinical infection can compromise efficient meat and egg production as well as animal welfare [2-4]. Four species, *Eimeria acervulina*, *E. maxima*, *E. necatrix *and *E. tenella*, are widely considered to pose the greatest threat to chicken production [5,6], although pathogenic examples have been reported for all seven species that infect the chicken (e.g. [7]). Traditionally eimerian infection has been diagnosed by microscopic examination of faecal or litter samples, looking for the environmentally resistant oocyst lifecycle stage, or post-mortem by lesion scoring [2,8]. While these traditional approaches can be highly effective, they can both suffer from a requirement for technical expertise, especially when identification of the infecting species is required [9]. Detection of subclinical infection can be particularly demanding in the absence of pathology. Advances in laboratory technologies have supported development of valuable new molecular diagnostics in response to these problems, including polymerase chain reaction (PCR), random amplification of polymorphic DNA PCR (RAPD-PCR) and DNA fingerprinting protocols [9-11], as well as quantitative PCR [12]. Although these technologies have been shown to be highly effective for use with *Eimeria*, requirements for relatively expensive specialist laboratory equipment or processing has limited their use in a manner similar to that described previously for many other pathogens [13,14]. Recognition of the tight economic margins in modern poultry production [15] and the impact of avian coccidiosis on poverty in many parts of the world, especially Asia [16], has highlighted a requirement for a panel of straightforward and sensitive, but cost effective, *Eimeria *species-specific diagnostic assays.

Loop-mediated isothermal amplification (LAMP) is a relatively simple technique which facilitates rapid DNA amplification with a high level of sensitivity [13,14,17-19]. Importantly, LAMP utilises the enzyme *Bst *DNA polymerase, which is active under isothermal conditions at a relatively high temperature and supports cost effective, rapid, target-specific amplification [18,19]. Briefly, LAMP is based upon an autocycling strand-displacement reaction using a set of four oligonucleotides which recognise six DNA sequences within the target genomic region and form a loop-structured amplicon. Additional loop primers may be added to improve amplification [18]. The efficiency and high yield of a LAMP reaction supports the use of intercalating dyes such as SYBR Green or hydroxynaphthol blue, enabling identification of a positive reaction with the naked eye [20,21]. LAMP assays have previously been developed for apicomplexan parasites including *Babesia orientalis*, *Cryptosporidium *species, *Plasmodium falciparum*, *Theileria parva *and *Toxoplasma gondii *[14,17,22-24]. In the absence of an easily accessible bloodstream form the robust nature of the eimerian oocyst has until recently hindered development of LAMP protocols for use with the *Eimeria *species. DNA extraction protocols from *Eimeria *oocysts in faecal material or litter can be time consuming, risk contamination with faecal PCR inhibitors and require a range of laboratory equipment [25]. Following the adaptation of a simple protocol for the isolation of DNA from forensic-type samples [26] for use with intracellular *Eimeria *in mucosal tissue we have developed a panel of LAMP assays specific for each of the seven recognised *Eimeria *species which infect the chicken.

## Methods

### Parasites and animals

Total genomic DNA extracted from the Houghton (H) strains of *E. acervulina*, *E. brunetti*, *E. maxima*, *E. mitis*, *E. necatrix*, *E. praecox *and *E. tenella *where used throughout these trials, all of which were isolated at the Houghton Poultry Research Station (UK). All parasites were propagated *in vivo *in three to seven week old Light Sussex chickens under specific pathogen free (SPF) conditions at the Institute for Animal Health and purified using established methods [27]. Genomic DNA was extracted from mechanically disrupted purified oocysts (i.e. free of bacterial and host cell contamination) and chicken blood as described previously [12].

### *Eimeria *species-specific LAMP oligonucleotide design

*Eimeria *genomic sequences identified previously during the development of a panel of *Eimeria *species-specific quantitative PCR assays were chosen for use in this study [12]. Candidate LAMP oligonucleotide sets including F3, B3, FIP and BIP were designed for each target using Primer Explorer V4 http://primerexplorer.jp/e/ Table 1) using default parameters with the exception that each component oligonucleotide melting point was targeted within the range 60-64°C. Loop primers LB and LF were designed independently using Pimer3 [28] using default parameters.

**Table 1:** Oligonucleotides developed for *Eimeria *species-specific LAMP PCR.

Species	Target	**Acc. no**.	Primer ID	Sequence (5' - 3')
*E. acervulina*	Ac-AD18-953^a^	AY571534	Eac_F3	CCTAACATTTCGCTTCACGGAC
			Eac_B3	ATGAGCAAGTGGAACACCTTG
			Eac_FIP	AGAGCACAGTGGCAGTGC-AGCAGACAGCATGGCTTACCT
			Eac_BIP	GAAGACCCTCTGAAGAACGGA-CCTTCTCACCGCTTACCGG
			Eac_LB	TAAGGTTACACCCGTGGAGG
			Eac_LF	GCCATGCACAAAGCGACTT
*E. brunetti*	Br-J18-626^a^	AY571556	Ebr_F3	GGCCATCAAGTTCCATGAGC
			Ebr_B3	TCAACCTCCTGAGTGTGGTT
			Ebr_FIP	GAAAATGCCTTCGTAGCTGCT-GCTGGGTACGGAGCGTCTT
			Ebr_BIP	TACTTCCTAGGATCCATCCTCGC-AGTTTCGCTGCCGCCTC
			Ebr_LB	GAAACGCTCGAACATGGC
			Ebr_LF	CTTCTCCACAGACCCAGAGGT
*E. maxima*	*Em*MIC1	M99058	Ema_F3	ACTACGGAAAAGTGCGTAGCT
			Ema_B3	CCTTCCTCCCTTCTGAAAACTG
			Ema_FIP	GAGTCACTGCTGATGTACCAAAAG-GAACTATGCCGCTTTCCCCTG
			Ema_BIP	AGAATGCGGATTTGTTAGCAGC-AGCAAGTCCAAGGTGTGTGTA
			Ema_LB	CAAGCCTACGCGGACATC
			Ema_LF	TTATGCAGCTGGGTCAACG
*E. mitis*	Mt-A09-716^a^	AY571506	Emi_F3	ACGATAGCCAAGACACGTAAGG
			Emi_B3	CCCCGTGATAAGAGTAGGAACA
			Emi_FIP	CGCGGGTCGTGAGATTTAAATTAT-GGAAGATCAGGACGGGCACT
			Emi_BIP	GTTTCAGTTGATGAACAAGCGAGA-TGCGCCTCTAGAATCAAGACG
			Emi_LB	TCCATGCATCCCCTTGTT
			Emi_LF	CGTGGGCACAGATTGATTC
*E. necatrix*	Nc-AD10-702^a^	AY571565	Ene_F3	TGGCTTTCCCGCGTACC
			Ene_B3	CGGCCCAACACAAAGACTG
			Ene_FIP	CGCTTGAGTTTTAAGCTATGCACA-GACCCAAGCAGCTCACCAA
			Ene_BIP	CGCCATGCCATTCAATGAACG-GAGGCATACCGGCGTTGTC
			Ene_LB	GTCTGTAACTTGGGACGTTGT
			Ene_LF	GAACAGCCGGAGCCTCTC
*E. praecox*	Pr-A09-1108^a^	AY571603	Epr_F3	GCCCTTGTATGTTGCTGTTTCT
			Epr_B3	GCGCACGAATCTGAATCACAC
			Epr_FIP	ATCTCCTCAAAGACTTTCGCGTA-GCGCTTGGCTATATCCATAGG
			Epr_BIP	GCTCTCGTGGCATACTTGC-GCCAGGAGCCACTGATTGT
			Epr_LB	GAATAGCATTGCCAGGTGG
			Epr_LF	GTCCACTGTCATTAATATTGCTGC
*E. tenella*	Tn-E03-1161^a^	AY571629	Ete_F3	GCTTGTGAAGGTCAGCGTG
			Ete_B3	GCTGAGTCCATACGTACTTCCT
			Ete_FIP	GCCACTGCTATGGAAAGTCACAC-CATAACTGGCATGCAGGGGT
			Ete_BIP	GTTTGGCCCGAAAGTTGTGAAGA-CGTCAGAAATTGCTGCCCAAT
			Ete_LB	CGCATGTGCAGTTGAAGACA
			Ete_LF	CCAAATGTATCTGCTAGTTATATTAACAAG

^a^*Eimeria *species-specific SCAR markers [30].

### LAMP F3 and B3 primer specificity tested by PCR

Each outer LAMP primer pair (F3 and B3; Table 1) was initially tested for *Eimeria *species specificity using PCR with BIO-X-ACT Short DNA Polymerase (Bioline Ltd.). Each PCR reaction contained 25 ng template DNA, 20 pmol of the relevant F3 and B3 primers, 0.5 U *Taq *polymerase, 10 mM Tris-HCl, 1.5 mM MgCl_2_, 50 mM KCl and 0.2 mM dNTPs. Standard cycle parameters were 1 × (5 min at 95°C), 30 × (30 sec at 95°C, 30 sec at 58°C, 1 min at 68°C) and 1 × (10 min at 70°C). Post-amplification PCR products were resolved by agarose gel electrophoresis. PCR fragments of interest were gel excised and purified (minelute gel purification, Qiagen), cloned using pGEM^®^-T Easy (Promega) in XL1-Blue *Escherichia coli *(Stratagene) and miniprepped (Qiagen) as described by the respective manufacturers. Candidate plasmids identified by *Eco *RI digestion and gel electrophoresis were sequenced (GATC Biotech) and analysed using CLC Main Workbench version 5.7.1 (CLC Bio, Denmark).

### LAMP assay

Each LAMP reaction was performed in a final volume of 25 μl containing 8 U *Bst *DNA polymerase (large fragment; New England Biolabs) in 1 × ThermoPol Reaction buffer (20 mM Tris-HCl, 10 mM KCl, 10 mM (NH_4_)_2_SO_4_, 2 mM MgSO_4 _and 0.1% Triton X-100; New England Biolabs) supplemented with 2 mM MgCl_2_, 1 M betaine and 400 μM of each dNTP. LAMP oligonucleotides FIP and BIP (40 pmol), LB and LF (20 pmol) and F3 and B3 (5 pmol), were added together with 10 ng or 2 μl template DNA (assay screening or sensitivity/field sample testing respectively). Each reaction was incubated at 62°C for 30 min and then 80°C for 5 min to terminate the reaction using a standard heat block or water bath.

### LAMP product detection

Positive LAMP results were identified by the addition of 120 μM hydroxynaphthol blue to each test reaction pre-incubation. At the conclusion of the incubation the colour of each reaction was assessed by eye under indoor light where negative results were differentiated from positive by a colour change from violet to sky blue [20,21]. LAMP reaction products were also resolved electrophoretically (2% Invitrogen agarose in 1 × TBE buffer) and visualized using SafeView nucleic acid strain (NBS biologicals).

### *Eimeria *species-specific LAMP specificity and sensitivity

Each candidate LAMP assay was initially screened for specificity against a panel of genomic DNA representing each of the seven *Eimeria *species that infect the chicken as well as chicken genomic DNA (background control). Where assay screening indicated species-specificity additional reactions were run using logarithmic genomic DNA dilution series in triplicate for the relevant *Eimeria *species only to establish sensitivity. DNA dilution series representing 10^5 ^to 10^0 ^parasite genomes per 2 μl were produced using glycogen (33 μg ml^-1^) as a carrier and validated by quantitative PCR as described previously [12].

### Field sample and DNA preparation

Intestinal sections collected from commercially produced broiler chickens reared under an extensive pasture-based system in Virginia, USA, were used as field samples to test the panel of LAMP assays. One bird was randomly selected from each of ten pens containing 50-60 birds for post mortem at 28 days of age. Intestinal sections of ~5 cm in length were collected from the duodenum, jejuno-ileum (spanning Meckel's diverticulum) and caeca. Samples were stored in RNAlater at -20°C after a period of stabilisation in the ambient conditions as described by the manufacturer (Ambion) and transported to the UK under IAPO importation licence. Subsequently, cells from ~1 cm^2 ^of the mucosal layer were scrapped free using either the edge of a sterile glass microscope slide or an ethanol/flame sterilised scissor blade and placed in a sterile 1.5 ml microcentrifuge tube containing (i) 100 μl sterile Tris-EDTA (TE) buffer or (ii) 100 μl 10% (w/v) Chelex 100 resin (Bio-Rad) in sterile TE buffer. Each sample was shaken vigorously for 1 min prior to incubation in a boiling water bath for 10 min. After boiling each sample was left to cool at the ambient temperature for 1-2 min before microcentrifugation at top speed (~10,000 g) for 1 min. Two microlitres of the resulting supernatant, containing the majority of the sample genomic DNA separated from the sample protein and Chelex 100, were assayed in each test LAMP reaction using genomic DNA recovered previously from uninfected SPF chicken mucosa as a negative control [29].

Additionally, each field sample was tested for the presence of *Eimeria *species parasites by traditional lesion scoring at the time of post mortem and by *Eimeria *species-specific multiplex PCR as described elsewhere [8,30]. Template genomic DNA for the multiplex PCR was extracted from an ~20 mg portion of each RNAlater preserved tissue section using the Qiagen Allprep DNA/RNA mini kit as described by the manufacturer.

### Ethics statement

This study was carried out in strict accordance with the Animals (Scientific Procedures) Act 1986, an Act of Parliament of the United Kingdom. All animal studies and protocols were approved by the Institute for Animal Health and Royal Veterinary College Ethical Review Committees and the United Kingdom Government Home Office under the project licence 30/2545.

## Results

### LAMP specificity

**Figure 1:**
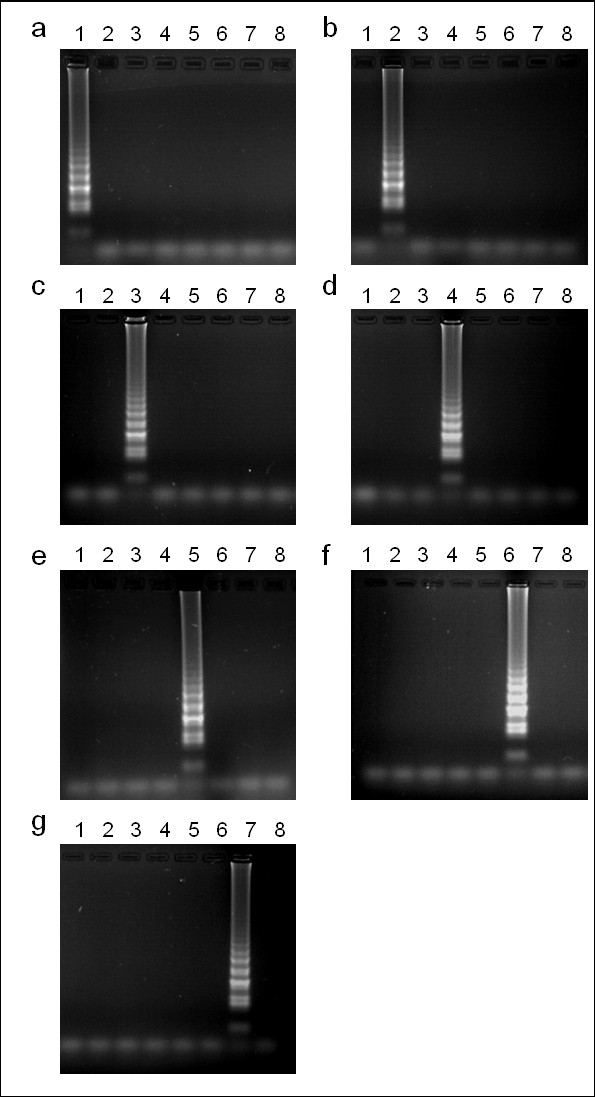
***Eimeria *LAMP assay species-specificity**. Electrophoresis of LAMP products amplified from a DNA panel representing *Eimeria acervulina *(lane 1), *E. brunetti *(2), *E. maxima *(3), *E. mitis *(4), *E. necatrix *(5), *E. praecox *(6), *E. tenella *(7) and the host (chicken, 8) as a background control. Panels represent LAMP assays specific for *E. acervulina *(a), *E. brunetti *(b), *E. maxima *(c), *E. mitis *(d), *E. necatrix *(e), *E. praecox *(f) and *E. tenella *(g).

Preliminary PCR screening using a DNA panel which included the seven *Eimeria *species that infect the chicken, in addition to chicken DNA as a background control, suggested that the outer primers designed for each LAMP assay were species-specific (F3 and B3; data not shown). Subsequent LAMP screening found each assay to be specific for the relevant target species when visualised using electrophoresis (Figure 1) or hydroxynaphthol blue.

### LAMP sensitivity

**Figure 2:**
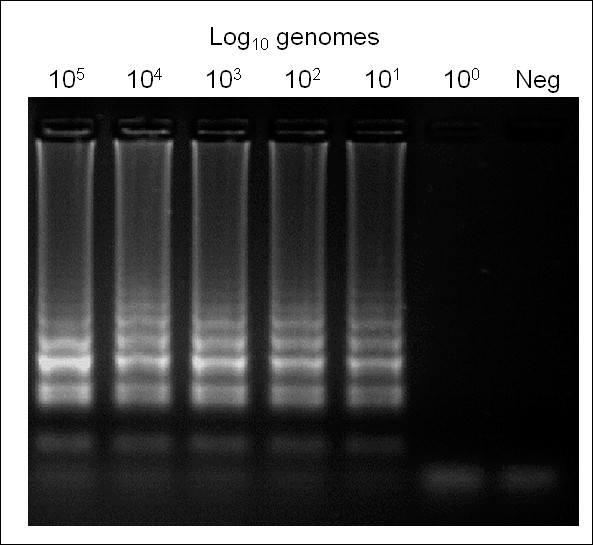
***Eimeria *species-specific LAMP assay sensitivity using *Eimeria tenella *as an example**. Neg = no template negative control.

LAMP using pure parasite genomic DNA dilution series revealed reproducible amplification from 10^1 ^genome copies but not 10^0 ^for all seven assays using electrophoresis (Figure 2) or hydroxynaphthol blue. Similarly, all seven species assays retained efficacy up to and including 10^5 ^genome copies, the highest concentration tested.

### Field sample testing: lesion scoring, LAMP and PCR compared

**Figure 3:**
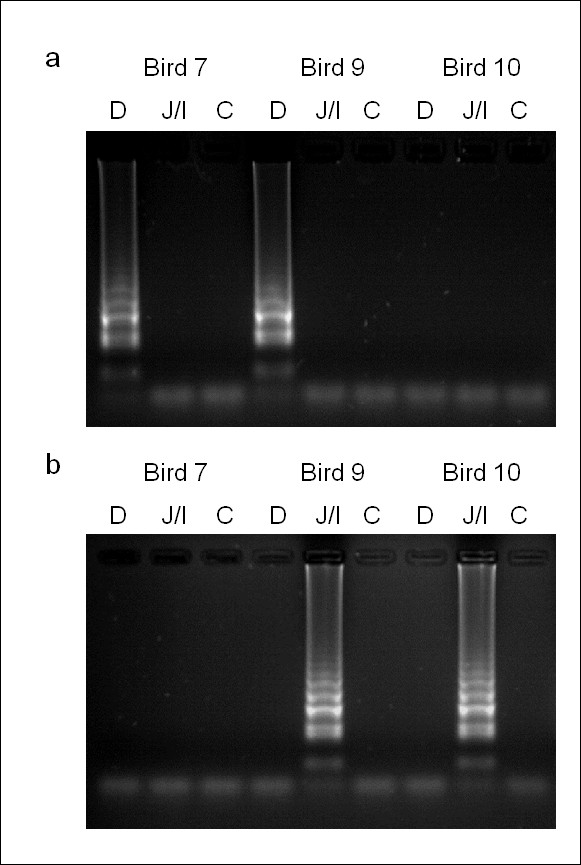
**LAMP diagnosis of eimerian infection from three field sample examples**. D = duodenal sample, J/I = jejuno-ileal sample, C = caecal sample. (a) *Eimeria acervulina *LAMP assay (b) *Eimeria maxima *LAMP assay.

**Figure 4:**
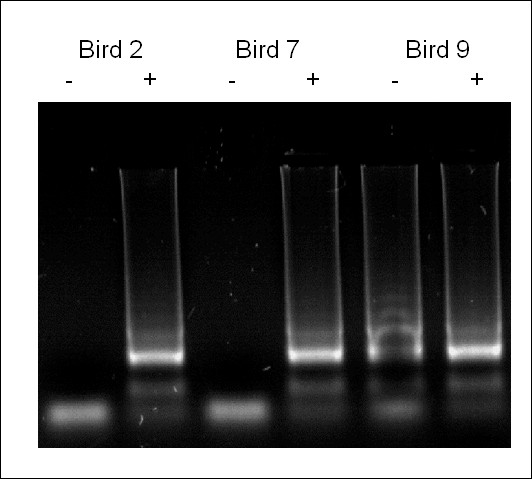
**The importance of Chelex 100 during field sample DNA preparation**. *Eimeria acervulina *LAMP of all three duodenal samples found to be positive for *E. acervulina *by lesion scoring and multiplex PCR. Samples prepared in the absence (-) or presence (+) of Chelex 100.

A total of ten randomly selected birds, each representing a separate pen, were humanely culled and subjected to post mortem. Lesion scoring identified low-grade *E. acervulina *and *E. maxima *infections in three and two birds respectively (Table 2). *Eimeria *species-specific multiplex PCR obtained identical results from high quality template DNA prepared using the Qiagen Allprep DNA/RNA mini kit (Table 2). Application of each LAMP assay using DNA extracted by boiling in the presence of Chelex 100 achieved results consistent with those obtained by lesion scoring and multiplex PCR, in addition to one further sample positive for *E. maxima *(Table 2, Figure 3). The inclusion of 10% (w/v) Chelex 100 in the LAMP field sample preparation protocol was essential to obtaining reproducible results that were consistent with the other diagnostic tests examined (Figure 4).

**Table 2:** LAMP diagnosis of eimerian infection from field samples compared with lesion scoring and multiplex PCR.

Pen no.	Lesion score^1 ^(species)				PCR^2^			LAMP	
			
	Duodenum	Mid-intestine	Caecae	Duodenum	Mid-intestine	Caecae	Duodenum	Mid-intestine	Caecae
1	-	-	-	-	-	-	-	-	-
2	1 (Eac)	-	-	Eac	-	-	Eac	-	-
3	-	-	-	-	-	-	-	-	-
4	-	-	-	-	-	-	-	-	-
5	-	-	-	-	-	-	-	Ema	-
6	-	-	-	-	-	-	-	-	-
7	1 (Eac)	-	-	Eac	-	-	Eac	-	-
8	-	-	-	-	-	-	-	-	-
9	1 (Eac)	2 (Ema)	-	Eac	Ema	-	Eac	Ema	-
10	-	1 (Ema)	-	-	Ema	-	-	Ema	-

Underlined entries were only detected using LAMP.

^1^Lesion scoring performed as described by Johnson and Reid [8]. ^2^Multiplex PCR performed as described by Fernandez et al [30].

## Discussion

*Eimeria *parasites can cause severe disease in poultry and even subclinical disease can incur economic loss [2,4]. Despite the development of several sensitive molecular tools the diagnosis of avian coccidiosis is still primarily achieved using traditional techniques including lesion scoring and faecal microscopy. Although robust, such techniques require expertise, can be time consuming and prove subjective when diagnosis is required to the species level [31]. Given these limitations it is likely that subclinical disease is under-diagnosed and that the true prevalence of *Eimeria *species parasites is unknown in much of the world. If molecular diagnostics are to address these problems, issues including equipment cost and availability of facilities will need to be resolved. Recognition of the influence of coccidiosis on global poultry production, as well as its impact on poverty in much of the world [15,16,32], has stimulated the search for reliable, sensitive and most importantly cheap new diagnostics.

LAMP has been widely recognised as a straightforward cost effective diagnostic tool with excellent reproducibility and sensitivity for nearly a decade and several assays targeting apicomplexan parasites have been described [14,17,22-24]. For the *Eimeria *species a limiting factor has been the ability to access the genomic DNA template. The environmental oocyst stage is the most readily accessible phase of the lifecycle but genomic DNA extraction from oocysts remains challenging in the absence of a laboratory, at least for those species which infect birds. The ability to extract eimerian DNA of a quality suitable for LAMP using equipment no more specialised than a microcentrifuge and a water bath now promotes the wider use of molecular biology in coccidial diagnostics. Similarly, the use of hydroxynaphthol blue or an equivalent indicator to use LAMP as a colorimetric assay removes the requirement for electrophoresis and provides an instant result [20,21]. While preserved tissue samples were used in the field trial described here there is no requirement for preservation. Supplementary LAMP tests using fresh, unpreserved tissues collected during standard parasite passage have demonstrated equal efficacy (data not shown).

The inclusion of Chelex 100, a chelating agent widely used in DNA extraction protocols for PCR, was found to be valuable in the preparation of DNA from unwashed and unpreserved avian mucosal cells for LAMP. The precise function of Chelex in the successful preparation of DNA for PCR remains unclear, although its sequestration of divalent heavy metals is likely to reduce PCR inhibition [26]. While LAMP is considered to be less sensitive to inhibitors than PCR [33] it is clear that inhibition was detected here, although this might have been a result of degradation during DNA preparation rather than genuine LAMP inhibition [26].

The choice of genomic template influences the sensitivity and specificity of a LAMP or PCR assay. Standard and quantitative PCR assays targeting *Eimeria *have used multicopy sequences including the internal transcribed spacer (ITS) regions and the 5S rDNA to improve sensitivity [29,34], although the relevance of sequence polymorphism has been highlighted [12]. Initially we designed LAMP oligonucleotide sets targeting the ITS-1 region of each *Eimeria *species, but found incomplete species-specificity to be an occasional problem when working with archived samples (data not shown). Our decision to target the sequences used previously in the development of an *Eimeria *species-specific qPCR panel reduced the sensitivity of each assay to between one and ten genome copies, which was comparable to the qPCR [12], but provided the necessary specificity. Knowledge that eimerian lifecycles feature multiple rounds of replication and several distinct stages, most of which contain ~14 or more genomes when mature, suggest such a sensitivity range is appropriate [35]. Indeed, LAMP was found to be a more sensitive diagnostic of eimerian infection than lesion scoring or non-quantitative PCR. In addition to species-specificity and sensitivity a reliable diagnostic assay also requires a lack of intra-specific variation in order to avoid false negative results. Although the nature of the LAMP targets is unknown for six of the *Eimeria *species tested (the exception is *E. maxima*, where the microneme protein 1 coding sequence was used), importantly parallel PCR and/or sequencing described previously support the existence of a suitable level of conservation in all isolates tested to date [11,12].

## Conclusions

The development of a panel of technically straightforward, fast and inexpensive LAMP assays suitable for the diagnosis of eimerian infection introduces a valuable new tool for use in poultry husbandry. Further validation using a wider range of field samples containing additional *Eimeria *species from different geographic regions and production systems will complement the studies described here.

## Authors' contributions

CB, RP and DB carried out the laboratory studies. RP carried out the field studies. VV contributed to preparation of the manuscript. DB designed and coordinated the study, and led preparation of the manuscript. All authors have read and approved the final manuscript.
